# Supplementary material for: Conditional chemoconnectomics (cCCTomics) as a strategy for efficient and conditional targeting of chemical transmission
Source: eLife. 2024 Apr 30;12:RP91927. doi: 10.7554/eLife.91927 (PMC11060718; doi:10.7554/eLife.91927)
Supplement: Figure 3—source data 3. [file elife-91927-fig3-data3.zip › Figure 3 source data3 part 2.zip!/Figure 3-Source data 3-part2/1062-13-CRISPResso_Report_jt28fhr9.zip!/CRISPResso_on_1062_15/1c.Alignment_barplot.pdf]

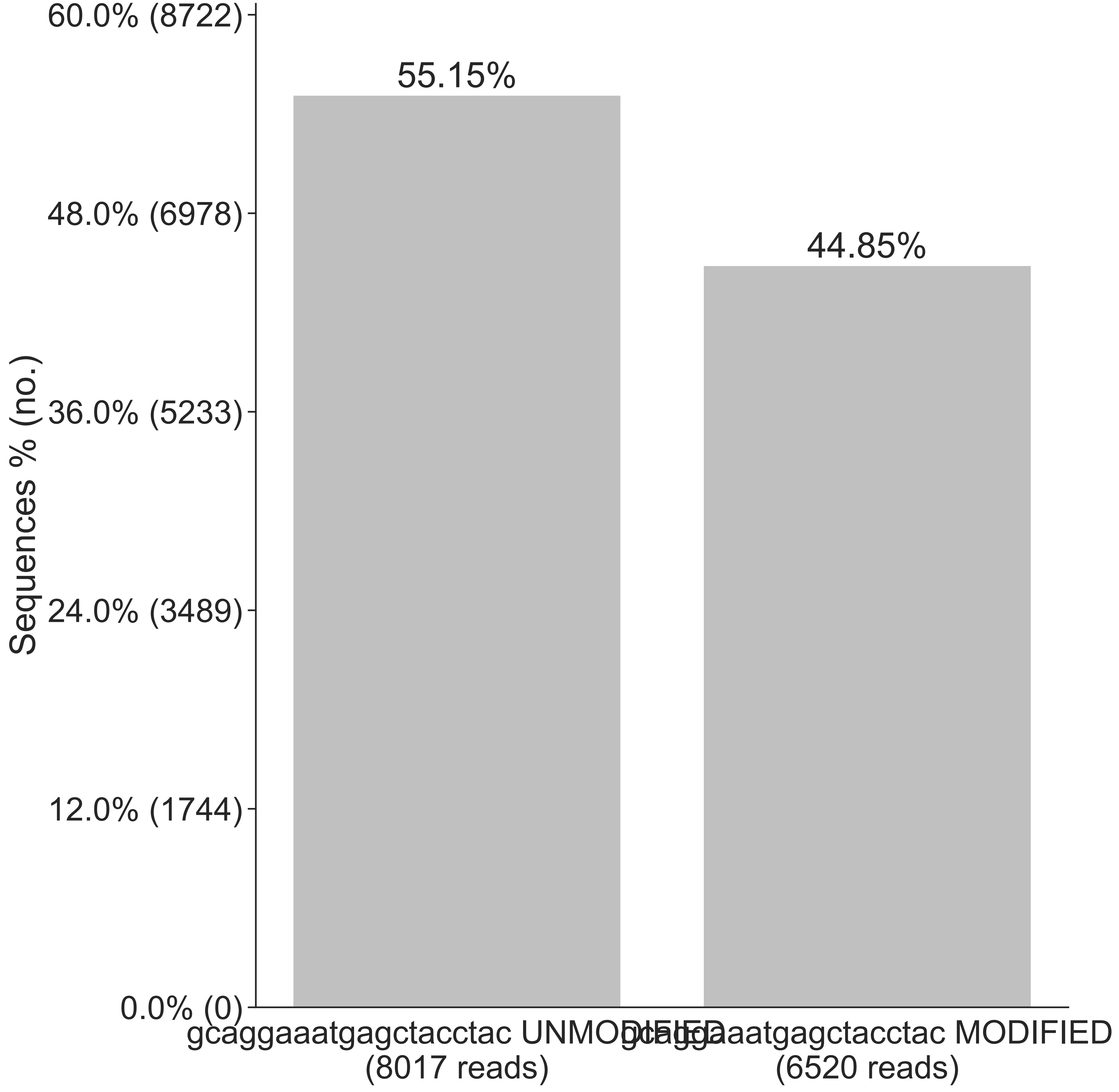

Sequences % (no.)
60.0% (8722)
48.0% (6978)
36.0% (5233)
24.0% (3489)
12.0% (1744)
0.0% (0)
55.15%
44.85%
gcaggaaatgagctacctac UNMODIFIED
(8017 reads)
gcaggaaatgagctacctac MODIFIED
(6520 reads)
